# Néel Tensor Torque in Polycrystalline Antiferromagnets

**DOI:** 10.1002/adma.202506462

**Published:** 2025-08-29

**Authors:** Chao‐Yao Yang, Sheng‐Huai Chen, Chih‐Hsiang Tseng, Hsiu‐Hau Lin, Chih‐Huang Lai

**Affiliations:** ^1^ Department of Materials Science and Engineering National Tsing Hua University Hsinchu 300044 Taiwan; ^2^ Department of Materials Science and Engineering National Yang Ming Chiao Tung University Hsinchu 300093 Taiwan; ^3^ Center for Emergent Functional Matter Science National Yang Ming Chiao Tung University Hsinchu 300093 Taiwan; ^4^ Department of Physics National Tsing Hua University Hsinchu 300044 Taiwan; ^5^ College of Semiconductor Research National Tsing Hua University Hsinchu 300044 Taiwan

**Keywords:** field‐free switching, neel tensor torque, physically unclonable functionality, spin‐orbit torque

## Abstract

Antiferromagnets (AFMs) offer exceptional promise for next‐generation spintronic devices due to their ultrafast dynamics and resilience to external perturbations. However, while single‐crystalline AFMs have been capable of being electrically manipulated, controlling polycrystalline AFM spins remains a major challenge due to their aperiodic nature. In this work, a Néel tensor is introduced as a rank‐two symmetric tensor that statistically captures the spin correlations in polycrystalline AFMs, a fundamental departure from the conventional Néel vector approach. Using machine learning techniques, hidden statistical patterns in AFM spin structures are extracted, and establish the Néel tensor torque, an emergent symmetry‐breaking mechanism at the FM/AFM interface. This torque enables field‐free spin‐orbit torque (SOT) switching in heavy‐metal/FM/AFM trilayers. Furthermore, it is experimentally demonstrated that the Néel tensor can be trained and memorized, allowing the system to retain its switching polarity–an unprecedented feature in AFM spintronics. This work unveils previously hidden statistical correlations in polycrystalline AFMs, bridging the gap between theoretical models and practical spintronic applications. The findings lay the foundation for non‐volatile, reconfigurable spintronic memory and neuromorphic computing, establishing the Néel tensor as a new degree of freedom for AFM‐based SOT switching.

## Graphical abstract

This work introduces a Néel tensor torque based on a rank‐two symmetric tensor capturing spin correlations in a polycrystalline antiferromagnet. It shows the Néel tensor can be shaped and reshaped through the spin‐orbit torque (SOT) technique, enabling field‐free SOT switching with a specific polarity of the adjacent ferromagnet. This discovery opens a new avenue toward reconfigurable spintronic memory utilizing polycrystalline antiferromagnets.

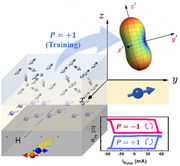

## 1 Introduction

Magneto‐resistive random‐access memory (MRAM) has emerged as a leading candidate for next‐generation non‐volatile memory, offering ultrafast switching, high endurance, and excellent scalability.^[^ ^1^, ^2^ ^]^ Its core functionality hinges on controlling magnetization states via different spin torque mechanisms. The field‐like torque is the most conventional, which arises from an external magnetic field or residual magnetization. However, Slonczewski's pioneering work introduced spin‐transfer torque (STT), wherein a spin‐polarized current directly exerts torque on a ferromagnetic (FM) layer, significantly enhancing energy efficiency and reducing power consumption.^[^ ^3^, ^4^ ^]^ More recently, spin‐orbit torque (SOT) MRAM has gained substantial attention, leveraging the spin Hall and Rashba effects to enable even more efficient magnetization switching.^[^ ^5^, ^6^ ^]^

Antiferromagnets (AFMs) have traditionally played a passive role in MRAM technology, primarily used to stabilize the FM layer via exchange bias interactions.^[^ ^7^, ^8^ ^]^ With a net‐zero magnetization in the bulk, AFMs were long thought to be magnetically inert components in spintronic architectures. However, the rapid rise of AFM spintronics has unveiled remarkable advantages over ferromagnets, including ultrafast spin dynamics, immunity to stray fields, and unconventional topological properties such as the anomalous Hall effect.^[^ ^9^, ^10^ ^]^ A major breakthrough in the field was the electrical switching of the Néel order in single‐crystal AFMs like CuMnAs,^[^ ^7^, ^11^, ^12^, ^13^ ^]^ demonstrating their potential as active elements in spintronic memory and logic applications. However, a fundamental limitation remains: most technologically relevant AFMs, such as IrMn and FeMn, are polycrystalline,^[^ ^14^, ^15^, ^16^ ^]^ lacking the long‐range periodicity required for conventional theoretical descriptions. Whether these polycrystalline AFMs can exhibit controllable spin‐orbit torque switching remains an open challenge.

To overcome this limitation, we introduce the concept of the Néel tensor, a novel mathematical framework capable of capturing the aperiodic spin configurations inherent to polycrystalline AFMs. Similar to how the Slonczewski torque arises from the interaction between magnetization and a spin‐current tensor, we propose that the FM/AFM interface hosts a novel tensor interaction beyond the conventional exchange‐bias effect. Unlike the conventional Néel vector, which is well‐suited for collinear AFMs, the Néel tensor provides a statistical representation of spin correlations in disordered and non‐collinear systems. By applying advanced statistical techniques from data science^[^ ^17^, ^18^ ^]^ we extract a rank‐two tensor that encapsulates the underlying spin interactions within polycrystalline AFMs. This new framework enables the definition of an emergent and previously unrecognized torque, the Néel tensor torque, which fundamentally differs from conventional spin torques.

The Néel tensor torque emerges as a novel symmetry‐breaking mechanism at the FM/AFM interface, enabling field‐free SOT switching. In trilayer heterostructures composed of a heavy metal/FM/polycrystalline AFM, we demonstrate that the Néel tensor can be trained to maintain a preferred orientation, effectively endowing the system with memory functionality. This finding marks a paradigm shift in AFM spintronics: rather than merely providing a static exchange bias, AFMs can actively participate in non‐volatile memory operations and introduce a new degree of freedom in device engineering.

In this study, we present direct experimental evidence for the existence of the Néel tensor torque at the FM/AFM interface in SOT switching. We further establish a comprehensive theoretical framework detailing the Néel tensor's properties and interactions, leading to a quantitative description of its torque dynamics. Finally, we demonstrate the training and retraining of the Néel tensor through controlled SOT switching cycles, showcasing its potential for reconfigurable spintronic memory applications. Our findings not only uncover previously hidden statistical correlations in polycrystalline AFMs but also establish a fundamentally new mechanism for AFM‐based spintronics. These insights pave the way for future advancements in non‐volatile memory technologies, artificial intelligence hardware, and next‐generation neuromorphic computing.

## 2 Results

### 2.1 An Unseen Torque at the FM/AFM Interface

**Figure 1:**
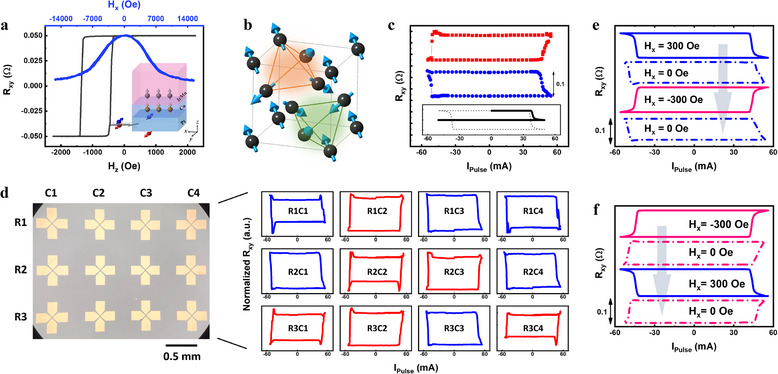
SOT switching of Pt/Co/IrMn trilayer with physically unclonable functionality (PUF). a) The Hall resistance *R_xy_* of the Pt/Co/IrMn trilayer versus the perpendicular field (*H_z_*) and longitudinal field (*H_x_*). The exchange bias is perpendicular without any noticeable longitudinal component. Inset: Schematic diagram of the SOT switching in Pt/Co/IrMn trilayer. b) Magnetic structure of AFM IrMn with the 3Q spin texture. Two tetrahedrons highlighted by orange and green stand for two kinds of 3Q configurations: spins at the corners of the tetrahedron pointing outward (orange) and inward (green). c) Field‐free switching curves (red and blue curves) of the as‐fabricated Pt/Co/IrMn devices with opposite switching polarity taken from two different devices. The switching curve (black) at the bottom shows the SOT switching taken from a device composed of a Pt/Co bilayer in the absence of *H_x_*. The dashed line in grey represents the SOT switching curve taken with an *H_x_* = 500 Oe. d) Optical microscope image of the 3 ×  4 array of SOT devices composed of a Pt/Co/IrMn trilayer and SOT switching curves of the as‐fabricated Pt/Co/IrMn devices in the absence of an external magnetic field, corresponding one‐to‐one with the devices shown in the optical microscope image at left. The switching polarities, clockwise and counterclockwise, are highlighted in blue and red, respectively. The switching curves were displayed in normalized *R_xy_* as a function of the pulse current (Ipulse) applied to each device to highlight the investigated array's polarity distribution. e) *H_x_*‐dependent SOT switching curves performed beginning with *H_x_* = −300 Oe and f) *H_x_* =   − 300 Oe. Vertical arrows in (e) and (f) represent the time sequences of measurements.

The discovery of an unexpected torque at the FM/AFM interface provides a foundation for understanding the Néel tensor torque introduced later. We first present key experimental findings in spin‐orbit torque (SOT) switching^[^ ^19^, ^20^, ^21^, ^22^ ^]^ to establish this phenomenon. **Figure** **1**a illustrates the investigated trilayer structure, consisting of Pt/Co/IrMn, where the bottom Pt layer generates a spin current via the spin Hall effect. The middle Co layer is a perpendicular ferromagnet with (111)‐oriented crystallographic texture, while the top IrMn layer is a polycrystalline AFM with a locally non‐collinear 3Q spin texture.^[^ ^23^, ^24^, ^25^ ^]^ To characterize the magnetic properties of the Pt/Co/IrMn trilayer, we measure anomalous Hall effect (AHE) curves under perpendicular (*​H_z_*, black) and longitudinal (*​H_x_*, blue) magnetic fields. The *R_xy_‐H_z_* curve in Figure 1a reveals a perpendicular exchange bias of ≈1000 Oe, while the centrosymmetric *R_xy_‐H_x_* curve indicates a robust anisotropic field of ≈1 Tesla in the Co layer, with no significant tilting along the longitudinal direction. These observations confirm the absence of in‐plane exchange bias, providing a solid foundation for analyzing the subsequent SOT switching behavior. Figure 1b illustrates the 3Q spin texture of IrMn, featuring two distinct tetrahedron configurations: spins located at the tetrahedron corners with moments pointing inward (green) and outward (orange). When IrMn is grown on Co with the (111) texture, the textured IrMn (111) is promoted in the perpendicular direction.

In conventional SOT switching,^[^ ^26^, ^27^ ^]^ an external longitudinal magnetic field (​*H_x_*) is typically required to break parity symmetry in the perpendicular direction via field‐like torque. As shown in the inset of Figure 1c, for a Pt/Co bilayer device, SOT switching under *H_x_* = 500 Oe (dashed line) results in complete magnetization switching. However, a typical curve to the demagnetization state is observed in the absence of *H_x_* (solid line) rather than a full switching. Remarkably, our measurements reveal that the Pt/Co/IrMn trilayer enables field‐free switching, as demonstrated in the main panel of Figure 1c. Two identical Pt/Co/IrMn devices exhibit complete field‐free SOT switching with a notable *R_xy_* change of ∼0.1 Ω, comparable to the *R_xy_‐H_z_* response in Figure 1a. A closer inspection of the switching behavior reveals an intriguing property: the switching polarity is not deterministic. As shown in Figure 1c, certain devices switch clockwise (blue) while others switch counterclockwise (red). To investigate this phenomenon further, we examine an array of 4 × 3 as‐fabricated Pt/Co/IrMn devices, shown in Figure 1d, and find that the switching polarities appear randomly distributed without an apparent pattern. This randomness suggests an intrinsic device‐dependent polarity that is set during fabrication. Setting aside the underlying mechanism for now, such intrinsic polarity provides a promising opportunity for implementing physical unclonable functions (PUFs) for hardware security applications.^[^ ^28^, ^29^ ^]^

Intriguingly, we find that this intrinsic polarity can be encoded and trained through field‐assisted SOT switching. Figure 1e presents a sequence of switching curves that elucidate how devices are trained. Initially, we apply an external longitudinal field *H_x_* = 300 Oe (solid blue line), which induces a deterministic switching polarity. The device retains its trained polarity once the external field is removed, as evidenced by the subsequent field‐free SOT switching (dashed blue line). We subsequently apply a reversed field of *H_x_* = −300 Oe, which successfully inverts the switching polarity (solid red line). Remarkably, after removing H_x_, the device continues to switch with the trained polarity, as evidenced by the dashed red line of Figure 1e. The results suggest that the device learned an intrinsic polarity once trained by a full SOT switching cycle in the presence of a magnetic field. Importantly, throughout these measurements, we carefully verify that no in‐plane exchange bias emerges (see Figure S1, Supporting Information). To further substantiate this finding, we perform the same field‐assisted SOT training on another as‐fabricated device, using *H_x_* = −300 Oe, and observe the same trend with a reversed intrinsic polarity, as shown in Figure 1f. A series of H_x_‐dependent SOT switching measurements, presented in Figure S2 (Supporting Information), indicates that a built‐in effective field is ≈70 Oe, aligned with the direction of the initial H_x_. This effective field reflects the internal symmetry‐breaking mechanism introduced for field‐free switching.

Although a strong perpendicular exchange bias field is present, as evidenced by the shifted hysteresis loop, the SOT switching curves exhibit no apparent shift. This is because the interfacial spins in IrMn are not static during SOT‐induced switching; instead, they dynamically reorient in response to the magnetization reversal of the adjacent Co layer.^[^ ^5^ ^]^ This interfacial adaptability effectively cancels the pinning effect during the switching process, resulting in a centrosymmetric SOT switching loop.

The observed deterministic field‐free switching suggests the existence of an unconventional torque at the FM/AFM interface. Several key parameters dictate the switching polarity, including the direction of the spin current (σ), the sign of the spin Hall angle (θ_SH_), and the applied *H_x_*, as captured by the following equation:

(1) $$P=\sigma \;\mathrm{sign}\left(H_{x}\theta_{\mathrm{SH}}\right)\;$$

where P = ±1 represents the switching polarity: +1 for counterclockwise switching (*H_x_* = −300 Oe, Figure 1e) and −1 for clockwise switching (*H_x_* = 300 Oe, Figure 1e) (see Figures S3 and S4, Supporting Information for further discussion). Notably, when *H_x_* is removed entirely, the switching polarity should become undefined in the absence of an additional symmetry‐breaking mechanism. However, our observations indicate a robust, field‐free SOT switching behavior with an intrinsic polarity. Given that we have ruled out the presence of in‐plane exchange bias, this phenomenon strongly suggests the emergence of an unseen type of spin torque at the FM/AFM interface—a mechanism that we later identified as the Néel tensor torque.

### 2.2 Néel Tensor Torque: A New Mechanism for Field‐Free Switching

The observed deterministic field‐free switching in the Pt/Co/IrMn trilayer demands an explanation beyond conventional spin torques. Since the system lacks an in‐plane exchange bias, and field‐like and Slonczewski torques alone cannot break symmetry in the absence of an external magnetic field, we propose an alternative mechanism based on the Néel tensor torque—a novel spin torque arising from statistical spin correlations in polycrystalline AFMs. Unlike traditional spin torques originating from vectorial interactions, the Néel tensor torque emerges from a rank‐two tensor interaction at the FM/AFM interface. This interaction, unique to polycrystalline AFMs, enables a new type of SOT switching.

### 2.3 Defining the Néel Tensor

**Figure 2:**
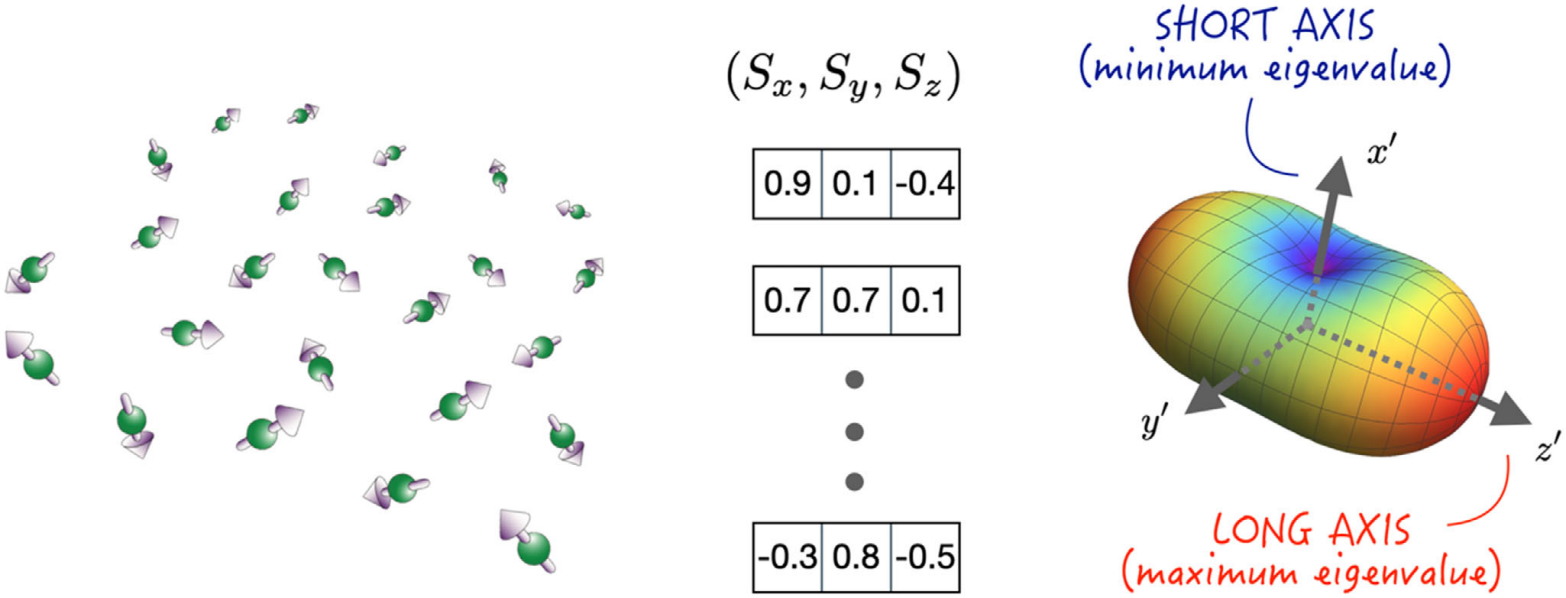
Extracting statistical patterns by the Néel tensor. In polycrystalline AFM, the orientations and positions of the spins are spatially random (the left figure), making the Fourier transform unsuitable for extracting statistical patterns. Instead, Principal Component Analysis (PCA) within the broader category of linear factor models proves useful. Each spin is treated as a 3D vector (the center figure) to train a neural network using the generalized Hebbian learning algorithm. PCA identifies statistical patterns (principal axes) ranked by frequency of occurrence, with the long axis representing the most frequent orientation and the short axis representing the least frequent (the right figure).

In polycrystalline AFMs such as IrMn and FeMn, the local spin ${\vec{s}}_{i}$ (where $i=1,2,\text{…},N_{s}$) exhibits spatial randomness, lacking the periodicity required for conventional first‐principles calculations. As a result, traditional methods struggle to capture the hidden spin correlations in polycrystalline AFMs. Different types of data require different algorithms to extract hidden statistical patterns. Learning from image processing, we employ machine learning techniques, specifically the linear factor model,^[^ ^30^, ^31^ ^]^ to extract statistical patterns from these aperiodic spin structures. For example, the aperiodic spin configuration composed of 25 spins, shown in **Figure** **2**, can be viewed as an ensemble of 3D vectors. Principal Component Analysis (PCA) can then be used to identify the dominant spin correlations and extract statistical patterns (principal axes) ranked by frequency of occurrence. Remarkably, an aperiodic spin configuration can be represented as a rigid body, with the long axis representing the most frequent orientation and the short axis representing the least frequent.

This approach allows us to decompose the spin structure into two key components: a residual spin vector ${\vec{S}}_{R}$ and a Néel tensor $\overset{↔}{N}$, which together provide a complete description of the local AFM spin environment:

(2) $${\vec{S}}_{R}\equiv \sum_{i=1}^{N_{s}}{\vec{s}}_{i}$$

(3) $$N^{\alpha \beta }\equiv \;\sum_{i=1}^{N_{s}}n_{i}^{\alpha }n_{i}^{\beta }=\left(\sum_{i=1}^{N_{s}}s_{i}^{\alpha }s_{i}^{\beta }\right)\;-S_{R}^{\alpha }S_{R}^{\beta }\;$$

where ${\vec{S}}_{R}$ represents the residual spin vector, capturing uncompensated spins primarily located at the FM/AFM interface, and is directly related to the exchange bias; $\overset{↔}{N}$ is the Néel tensor, encoding spin correlations within the AFM. The Néel tensor is a real, symmetric, and semi‐positive definite matrix, meaning it can be diagonalized into mutually orthogonal principal axes. Here, ${\vec{n}}_{i}={\vec{s}}_{i}-{\vec{S}}_{R}$ describes fluctuations relative to the residual spin ${\vec{S}}_{R}$, and the superscripts α, β  =  *x*, *y*, *z* correspond to different spin components. Note that, for disordered spin clusters, the cumulant expansion can be employed to systematically capture their statistical characteristics, order by order. The first order of the cumulant expansion is the residual spin ${\vec{S}}_{R}$, and the second order is the rank‐two Néel tensor $\overset{↔}{N}$. These are independent parameters to describe the statistical patterns in a disordered spin cluster. For strongly correlated AFMs (for example, spin glass), higher‐order statistical moments of the spin configurations can be incorporated using higher‐rank tensors, following the logic of cumulant expansions.

### 2.4 Néel Tensor Interaction and Visualization

The Néel tensor not only captures the spin structure of polycrystalline AFMs but also governs a new type of interaction. Conventionally, the exchange bias arises from the interaction between the residual spin ${\vec{S}}_{R}$ and the magnetization in an adjacent FM layer. However, in our system, where in‐plane exchange bias is absent, we propose that the Néel tensor $\overset{↔}{N}$ itself interacts with the FM magnetization.

Since interaction energy must be a scalar, tensor analysis suggests that the most straightforward interaction involves two inner products (tensor contractions in professional terminology) and is given by:

(4) $$U_{N}=\frac{1}{2}\lambda_{N}\;\vec{M}\cdot \overset{↔}{N}\cdot \vec{M}=\frac{1}{2}\lambda_{N}\;\left(M_{x}\;M_{y}\;M_{z}\right)\left(\begin{array}{ccc} N^{xx} & N^{xy} & N^{xz}\\ N^{yx} & N^{yy} & N^{yz}\\ N^{zx} & N^{zy} & N^{zz} \end{array}\right)\left(\begin{array}{c} M_{x}\\ M_{y}\\ M_{z} \end{array}\right)\;$$

where λ_*N*_ denotes the coupling constant of the Néel tensor interaction *U_N_*. When λ_*N*_ > 0, the Néel tensor's short axis (corresponding to the smallest eigenvalue) aligns with $\vec{M}$ to minimize the interaction energy; conversely, for λ_*N*_ < 0, the Néel tensor's long axis (associated with the largest eigenvalue) aligns with $\vec{M}$.

In collinear AFMs, the Néel vector, which coincides with the long axis of the Néel tensor, is typically perpendicular to the adjacent FM magnetization, suggesting a positive λ_*N*_. For polycrystalline AFMs, particularly those with non‐collinear spin textures, the full rank‐two Néel tensor is necessary to correctly describe the interactions. In the following, we would assume λ_*N*_ > 0 in our device, but the major conclusion does not rely on the sign of the coupling constant. Although the tensorial interaction may seem complex, similar forms appear in molecular interactions in liquid crystals^[^ ^32^ ^]^ and nuclear spin interactions in nuclear magnetic resonance,^[^ ^33^ ^]^ where well‐established theoretical techniques provide insight.

To gain an intuitive understanding, we visualize the Néel tensor interaction using spherical plots, where the magnetization $\vec{M}=M\;\hat{r}=M\left(\sin\theta \cos\phi ,\sin\theta \sin\phi ,\cos\theta \right)$ is expressed in the spherical coordinates,

(5) $$U_{N}=\left(\frac{1}{2}\lambda_{N}M^{2}\right)\;\hat{r}\cdot \overset{↔}{N}\cdot \;\hat{r}=\frac{1}{2}\lambda_{N}\;M^{2}\;u_{N}\left(\theta ,\phi \right)\;$$

The Néel tensor interaction *U_N_* is quadratic in the strength of the magnetization, and its angular dependence is fully captured by the reduced Néel tensor interaction $u_{N}\;\left(\theta ,\phi \right)=\hat{r}\;\cdot \overset{↔}{N}\cdot \hat{r}$, solely determined by the Néel tensor. In consequence, *u_N_* (θ,ϕ) provides the visualization of the Néel tensor interaction *U_N_*, or the Néel tensor $\overset{↔}{N}$ itself, making the Néel tensor interaction more intuitive and experimentally accessible.

**Figure 3:**
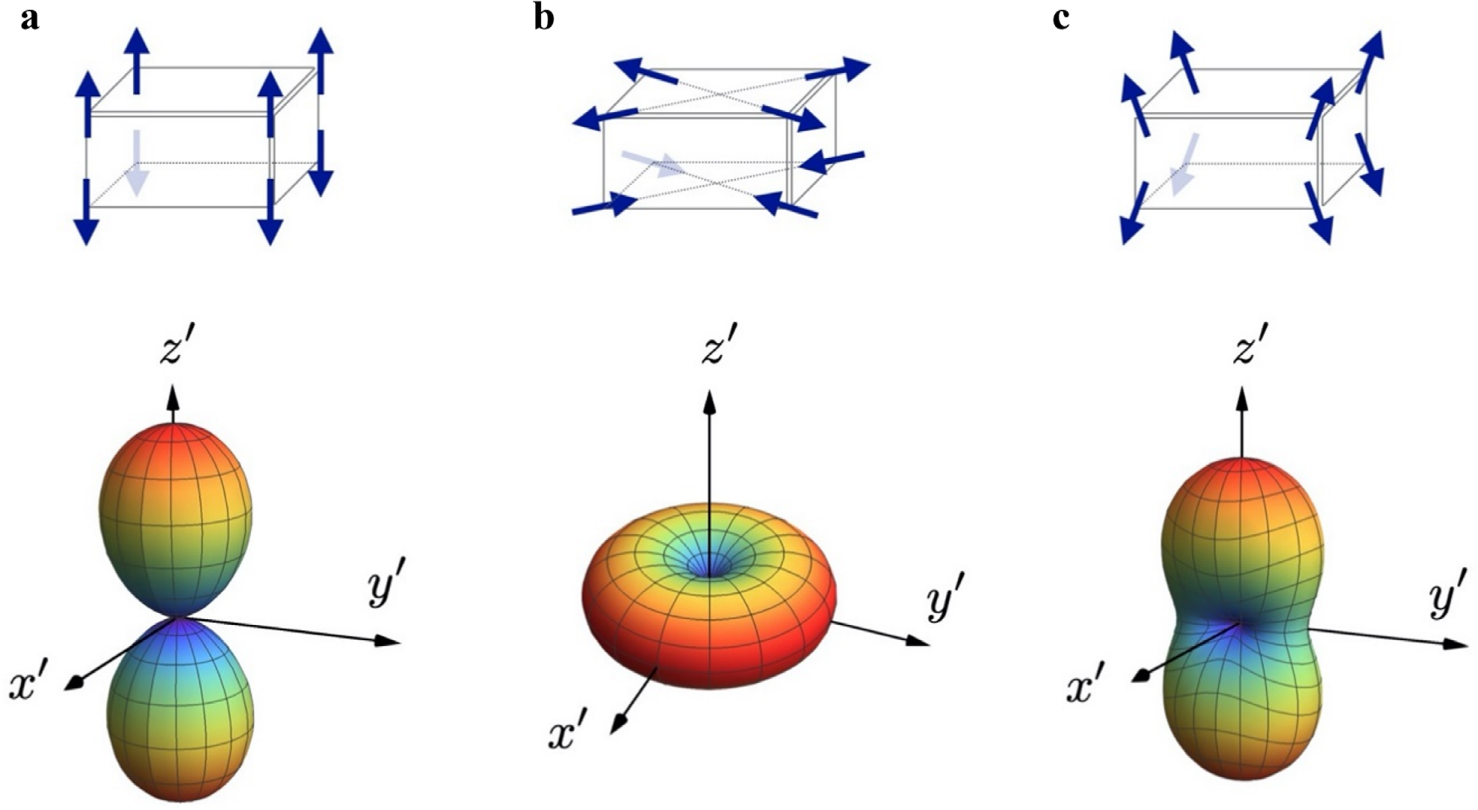
Visualizing the reduced Néel tensors. Taking an AFM domain with eight spins as a demonstration example, the spherical plot of the reduced Néel tensor interaction reveals the connection with the corresponding spin configurations. In the spherical plot, the value of *u_N_* (θ,ϕ) is represented as the distance to the origin. For visual aids, it is also represented by rainbow colors: the maximum value is represented by red color, while the minimum value is represented by blue color. a) When all spins are collinearly aligned along the perpendicular direction, *u_N_* (θ,ϕ) reaches its maximum (colored by red) along the *z*′‐axis while its minimum (colored by blue) occurs in the transverse plane. The dumbbell‐shaped aligned with the *z*′‐axis can be described by the Néel vector due to its axial symmetry along the long axis. b) However, a non‐collinear spin configuration is more challenging to comprehend. For simplicity, consider the planar spin configuration with zero components in the perpendicular direction. The maximum of *u_N_* (θ,ϕ) occurs in the *x*′ − *y*′ plane while its minimum lies in the *z*′‐axis. The spherical plot resembles the “donut” shape with degenerate maximum principal axes in the *x*′ − *y*′ plane. c) For general spin configurations with considerable non‐collinearity, the reduced Néel tensor interaction resembles the “dough” shape with three orthogonal principal axes. It is clear that merely a preferential axis (represented by the Néel vector) is insufficient to capture the full features of spin arrangements in an AFM domain.

AFM spin configurations can exhibit a variety of spatial arrangements, which fundamentally influence their interactions with adjacent FM layers. In the context of current AFM‐based spintronics, three primary types of AFM textures have been identified (**Figure** **3**a–c): (a) collinear AFM, (b) non‐collinear but co‐planar AFM, and (c) non‐collinear and non‐coplanar AFM. Each spin configuration carries zero net magnetization, yet they give rise to distinct statistical spin correlations, which can be effectively captured by the Néel tensor. To visualize the impact of these different spin structures on Néel tensor interaction, we present the reduced Néel tensor interaction at the bottom of Figure 3a–c, in which the rainbow contour reveals the strength of the interaction between the Néel tensor and an adjacent FM. Here, red regions denote stronger interactions, while blue regions represent weaker interactions. All spin configurations in Figure 3 carry zero magnetization, and they can be easily characterized by the Néel tensor with spin variances along three principal axes. From a statistical perspective, the long axis of the Néel tensor aligns with the largest spin variance, while the short axis corresponds to the smallest variance. This distinction is crucial because it dictates how the Néel tensor torque interacts with magnetization, leading to the symmetry breaking for field‐free SOT switching. As elaborated in Figure S5 (Supporting Information), this statistical framework allows the Néel tensor to generalize beyond the conventional Néel vector, offering a powerful tool for describing aperiodic spin configurations in polycrystalline AFMs.

### 2.5 Néel Tensor Torque and Its Role in Field‐Free Switching

The Néel tensor interaction not only defines a novel form of spin interaction but also generates a distinct type of torque on the magnetization. This torque, termed the Néel tensor torque, arises directly from the coupling between the FM magnetization and the statistical spin correlations captured by the Néel tensor. Unlike conventional spin torques, which typically stem from external fields or spin currents, the Néel tensor torque originates from the intrinsic symmetry properties of the polycrystalline AFM.

**Figure 4:**
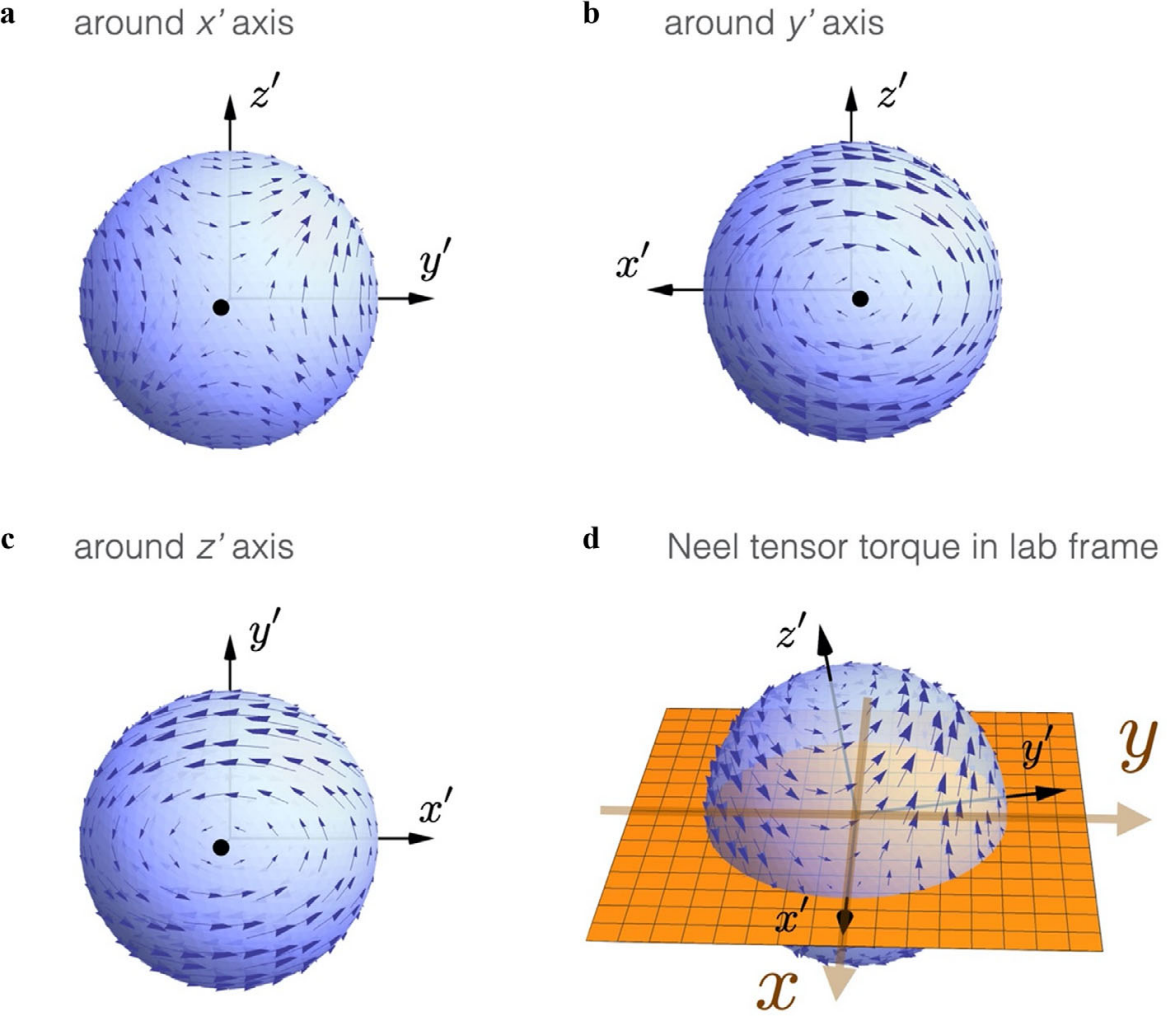
Néel tensor torque around principal axes. The deterministic Néel tensor torque vanishes when the FM magnetization $\vec{M}$ is along one of the principal axes of the Néel tensor. a) The torque distribution around the *x*′ axis (intermediate spin variance) is a saddle point. b) The torque distribution around the *y*′ axis (short axis) is a left‐handed neutral equilibrium. c) The torque distribution around the *z*′ axis (long axis) is a right‐handed neutral equilibrium. d) When the short axis of the Néel tensor is tilted in the 1st and the 3rd quadrants, the Néel tensor torque drives the magnetization $\vec{M}=\left(0,M_{y},0\right)$ upward or downward depending on the sign of *M_y_*.

Following the standard derivation for spin torques, the effective field associated with the Néel tensor interaction is obtained by computing the gradient of the interaction energy with respect to the magnetization. This leads to the expression for the Néel tensor torque:

(6) $${\vec{\tau }}_{N}=-\vec{M}\times \nabla_{M}\;U_{N}=-\lambda_{N}M^{2}\;\hat{m}\times \left(\overset{↔}{N}\cdot \hat{m}\right)$$

where $\hat{m}$ is the unit vector along the direction of $\vec{M}$. The Néel tensor torque involves both inner and outer products with magnetization, making it qualitatively distinct from the Slonczewski torque, as detailed in Figure S6 (Supporting Information). This distinction is crucial because it introduces an additional symmetry‐breaking mechanism beyond those provided by conventional spin torques. While the Néel tensor may result in an additional torque to the adjacent FM, its ability to induce field‐free switching depends on the alignment of its principal axes with respect to the laboratory frame. If the principal axes of the Néel tensor align with the coordinate axes of the lab frame, the resulting torque does not break the symmetry of spin‐orbit torque (SOT) switching, meaning that field‐free switching cannot be achieved, as illustrated in **Figure** **4**a–c On the contrary, if the principal axes of the Néel tensor are tilted relative to the lab frame, the Néel tensor torque introduces an intrinsic symmetry‐breaking effect that enables deterministic field‐free switching. Consider the scenario depicted in Figure 4d, where the short axis of the Néel tensor (denoted as the *y*′‐axis) is inclined within the first and third quadrants of the *y* − *z* plane (lab frame). This inclination modifies the interaction between the magnetization and the Néel tensor. When the magnetization points toward the positive *y*‐axis, $\vec{M}=\left(0,M_{y},0\right)$ with *M_y_* > 0, the Néel tensor torque drives the magnetization upward, as indicated by the arrows on the torque sphere. Conversely, when the magnetization points toward the negative *y*‐axis, $\vec{M}=\left(0,M_{y},0\right)$ with *M_y_* < 0, the Néel tensor torque drives the magnetization downward. This behavior indicates that the orientation of the Néel tensor's short axis plays a decisive role in determining the magnetization switching direction. Essentially, the Néel tensor torque acts as a built‐in symmetry‐breaking term. The Néel tensor torque (originating from the spin correlations in polycrystalline AFMs), together with the Slonczewski torque (generated by spin current), breaks mirror symmetry in a unidirectional manner, leading to deterministic field‐free switching with one‐fold symmetry. The intrinsic polarity of the switching process is dictated by the relative orientation of the Néel tensor's principal axes in the laboratory frame. This novel mechanism provides a robust strategy for engineering field‐free spintronic devices, offering advantages for next‐generation non‐volatile memory and logic applications.

Furthermore, when the short axis is tilted in the *y*′ − *z*′ plane, the torque configuration around the *x*′‐axis resembles a saddle‐point pattern, in contrast to the circular torque pattern around the *y*′‐axis. Consequently, if a current pulse is applied along the y‐axis, the resulting Slonczewski torque (driven by the spin current) initially pushes the magnetization toward the x‐axis (which coincides with the *x*′‐axis in this setup). However, the SOT switching cannot be achieved with a definite polarity because the vertical parity symmetry is not broken.

### 2.6 Training and Memory Effect in Néel Tensor Torque

The Néel tensor torque not only provides a mechanism for field‐free SOT switching but also introduces a training and memory effect, enabling the controlled manipulation of the intrinsic switching polarity. This phenomenon arises from the interaction between the magnetization and the Néel tensor's principal axes, allowing the system to learn and retain a preferred switching direction through an external training process. With the absence of an in‐plane exchange bias, the observed deterministic field‐free SOT switching can be attributed to the tilted Néel tensor, whose alignment governs the switching polarity. Interestingly, the learning and memorization process in the Néel tensor exhibits similarities to the glassy state behavior in Hopfield neural networks,^[^ ^35^ ^]^ emphasizing the critical role of polycrystalline AFMs in defining the functionality of the trilayer device.

**Figure 5:**
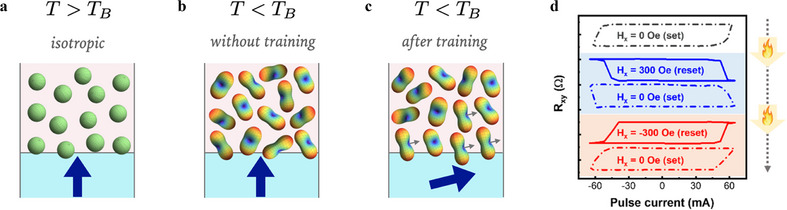
Néel glass in polycrystalline AFM. Schematic diagrams for the Néel tensors at different temperatures. a) Above the blocking temperature *T_B_* ∼150 °C, the spin arrangement is completely random, and the Néel tensors become isotropic spheres. b) When cooled below the *T_B_*, the Néel tensors start to take shape, but the principal axes are still randomly oriented. c) Going through the field‐SOT training, the short axis of the Néel tensors is aligned by the FM magnetization and provides the symmetry‐breaking spin torque. d) Experimental verification: The trained and retrained Néel tensors are verified experimentally through the polarity inversion of the field‐free SOT switching.

To understand how the Néel tensor maintains its orientation once trained, we examine the evolution of the Néel tensor across different temperatures. To encapsulate the spatial inhomogeneity, we can compute the Néel tensors corresponding to each AFM domain, as illustrated in **Figure** **5**. Above the blocking temperature (paramagnetic phase), the AFM correlations vanish, rendering the Néel tensors fully isotropic and randomly distributed, visualized as a collection of isotropic spheres (Figure 5a). When cooled below the blocking temperature *T_B_* (antiferromagnetic phase), the AFM correlations develop, and the Néel tensors acquire a distinct form (Figure 5b). However, due to the polycrystalline nature of the AFM, the principal axes of different Néel tensors remain randomly oriented with no global order. When the field‐assisted SOT training process is performed, the magnetization of the FM layer tilts during the pulse on, as depicted in Figure 5c. This tilting realigns the short axis of the Néel tensor through the coupling between FM and AFM, effectively encoding the preferred magnetization direction. Because polycrystalline AFMs exhibit multiple local energy minima, once the Néel tensor axes are trained into a particular alignment, they remain trapped in a local minimum and persist indefinitely. This phenomenon explains how the SOT device learns and memorizes the switching polarity through the field‐assisted SOT training process.

The memory of the SOT device can be both trained and erased, as demonstrated in Figure 5d. Heating the device above *T_B_* to 200 °C resets all Néel tensors to their isotropic state (Figure 5a), effectively erasing the intrinsic polarity. When the device is cooled below *T_B_* to room temperature and field‐assisted SOT training is performed with *H_x_* = 300 Oe (reset), the intrinsic polarity shifts to *P*  =   − 1 (clockwise switching) at the zero‐field state (set). If the device is reheated above *T_B_*, the memory is erased once again. Cooling it down and retraining it with *H_x_* =   − 300 Oe re‐establishes the polarity at *P*  =  1 (counterclockwise switching). This process confirms that the Néel tensor torque enables a tunable and reconfigurable polarity in field‐free SOT devices, distinguishing it from traditional exchange bias, which is typically fixed during fabrication.

### 2.7 Alternative Training Methods: Role of the Exchange Bias and Effective Field

**Figure 6:**
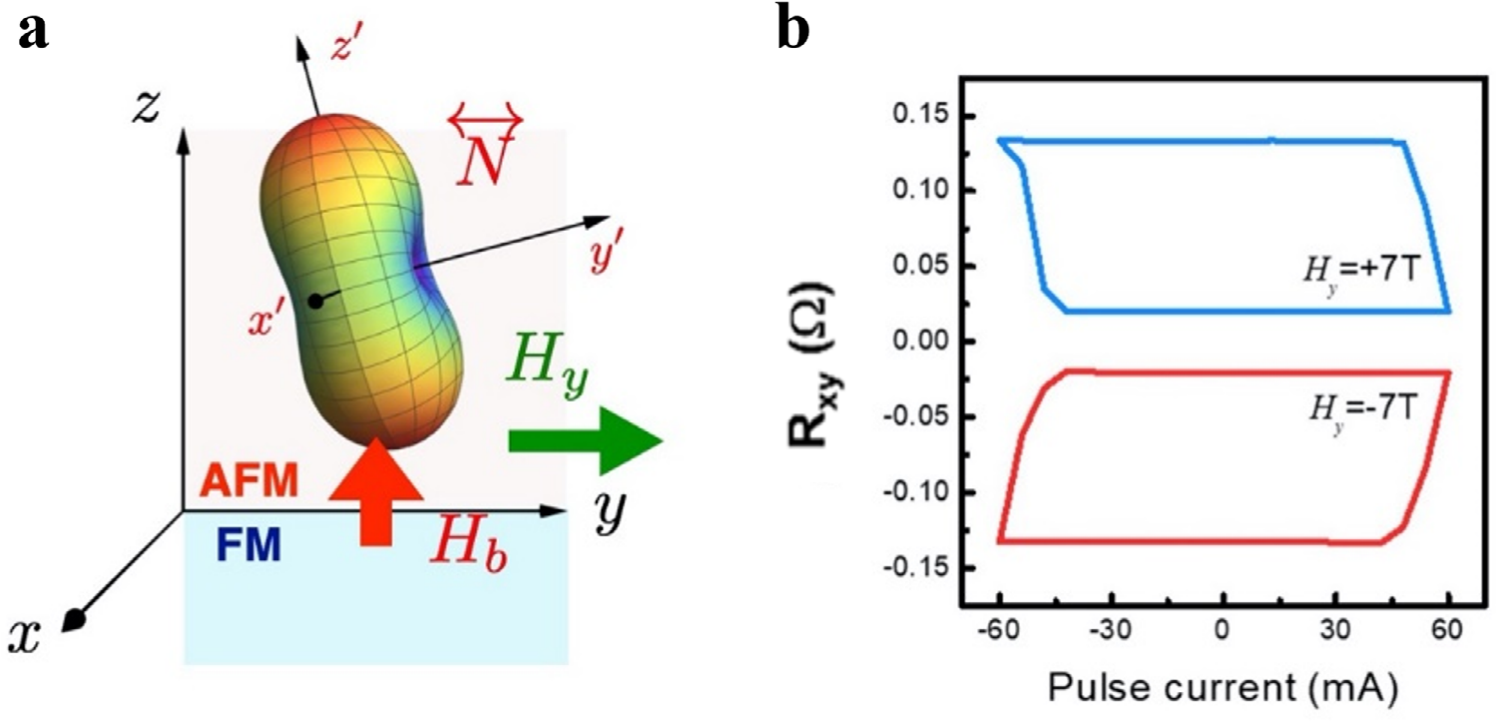
Setting the Néel tensor by a magnetic field. a) Combining the exchange bias ${\vec{H}}_{b}=\left(0,0,H_{b}\right)$ and the strong field $\vec{H}=\left(0,H_{y},0\right)$, the Néel tensor can be set by the effective field ${\vec{H}}_{e}ff$ in the *y* − *z* plane. b) The intrinsic polarity *P*  =   − 1 for *H_y_* =  7 T. The intrinsic polarity *P*  =  1 for *H_y_* =   − 7 T. These results validate the predicted intrinsic polarity for the zero‐field SOT switching.

Beyond field‐assisted SOT switching, we identify an alternative method to train the Néel tensor by combining perpendicular exchange bias and an external magnetic field, as depicted in **Figure** **6**a. By applying an external field along the *y*‐axis $\vec{H}=\left(0,H_{y},0\right)$, we observe a similar alignment effect. The applied field forces the magnetization in the FM layer to align in the same direction. When combined with the perpendicular exchange bias, the effective field is confined within the *y* − *z* plane, leading to the alignment of the short axis of the Néel tensor.

Our theoretical model predicts that the intrinsic polarity after training follows the sign rule:

(7) $$P=\sigma \;\mathrm{sign}\left(\theta_{\mathrm{SH}}\right)\cdot \mathrm{sign}\left(H_{y}H_{b}\right)$$

where σ represents the sign of the spin current direction, θ_SH_is the spin Hall angle, *H_y_* and *H_b_* are the applied and exchange bias fields, respectively. The detailed derivation is provided in Figure S6 (Supporting Information).

Our experimental results, shown in Figure 6, strongly support the theoretical predictions. Given that the spin current is injected from the bottom Pt layer, the system is characterized by σ  =   − 1, with a positive spin Hall angle in the Pt layer and a perpendicular exchange bias directed upward. Under these conditions, the sign rule simplifies to *P*  =   − sign(*H_y_*). When *H_y_* =  7 T is applied, the intrinsic polarity is set to *P*  =   − 1, aligning with theoretical expectations (Figure 6b). Reversing the training field to *H_y_* =   − 7 T shifts the trained polarity to *P*  =  1, demonstrating a fully reversible and controllable memory effect.

It is worth noting that although both field‐ and SOT‐training methods reorient the Néel tensor and enable robust, deterministic field‐free switching in Pt/Co/IrMn devices, accompanied by a perpendicular exchange bias, no measurable exchange bias is observed in the x–y plane after either training process. This confirms that the field‐free SOT switching is not driven by induced in‐plane exchange bias through pre‐training, but is instead governed by the Néel tensor torque.

## 3 Discussion

The Néel tensor framework introduced in this work provides a holistic representation of aperiodic spin configurations in polycrystalline AFMs, surpassing the conventional Néel vector approach. While our experimental results confirm that the Néel tensor can be trained and retrained using different external stimuli, an intriguing question remains: Why is the Néel tensor so easily trainable? In our polycrystalline IrMn film, the crystallographic direction is aligned perpendicularly, and the AFM coupling leads to the formation of 3Q tetrahedral spin arrangements. Theoretical calculations (Figure S7, Supporting Information) show that the ideal tetrahedral spin configuration results in a Néel tensor of zero. However, in real materials, domain‐domain interactions and microscopic spin‐dependent interactions introduce deviations from perfect tetrahedral order, resulting in a nonzero Néel tensor below the blocking temperature. Because the energy scale of domain‐domain interactions is relatively weak, the Néel tensor is highly responsive to external magnetic fields or magnetization alignment in adjacent FM layers. This property underpins the ease of training and reconfiguration observed in our experiments. The success of the field training is exciting and promising because the setting process does not involve the spin current and thus can be performed effortlessly.

A natural follow‐up question is: What behavior emerges in as‐fabricated devices that have not undergone explicit training via H_x_‐assisted SOT or the application of a strong magnetic field? As shown in Figure 1d, field‐free SOT switching can still occur, but the switching polarity appears random from device to device. This suggests that in pristine Pt/Co/IrMn stacks, the Néel tensor distribution is not completely random but exhibits partial local ordering. Such ordering likely arises from fabrication‐induced factors, including residual strain, grain orientation, and interfacial variations. A completely disordered Néel tensor ensemble would produce a vanishing net torque, precluding any field‐free switching. Thus, the observation of field‐free SOT switching with a random polarity implies the presence of partial symmetry‐breaking across the device. Moreover, within a single as‐fabricated device, multiple locally favorable Néel tensor orientations may coexist. This results in complex and non‐typical switching behaviors, such as that observed in device R2C2 in Figure 1d. At low current amplitudes, one orientation may dominate, defining the initial switching polarity. As the current increases, an alternate configuration may become energetically favorable, effectively reversing the direction of the Néel tensor torque and inducing a current‐driven polarity transition. This transition manifests as a change in switching polarity and increased stochasticity along the edges of the SOT loop. Importantly, such non‐typical switching behavior is eliminated after proper H_x_‐assisted SOT training, which aligns the principal axes of the Néel tensor uniformly across the device, as shown in Figure 5c. This reconfiguration enables robust, deterministic field‐free switching.

Although the Néel tensor can represent a statistical order parameter capturing spin correlations in polycrystalline AFMs, it is not as directly observable as conventional exchange bias. Several experimental strategies may indirectly or directly validate the presence and orientation of the Néel tensor. Clear evidence of anisotropic magnetoresistance (AMR) changes was reported in the previous work for the Pt/Co/IrMn trilayers, in which the longitudinal (transverse) resistance of the device increased (decreased) after the SOT switching.^[^ ^34^ ^]^ The AMR changes after SOT switching can indirectly verify the reorientation of the Néel tensor.

The direct detection of the Néel tensor could be pursued in future work via X‐ray magnetic linear dichroism (XMLD). The Néel tensor, being a second‐rank order parameter, can be probed via XMLD, which is sensitive to the anisotropic orientation of antiferromagnetic spin axes. In principle, angular‐dependent XMLD measurements, analogous to tomographic reconstruction in CT imaging, could provide a three‐dimensional mapping of the Néel tensor distribution. In contrast, X‐ray magnetic circular dichroism (XMCD) probes vector‐like components (such as uncompensated spins), allowing one to distinguish the Néel tensor torque from field‐like torques.

We have demonstrated the interaction of the Néel tensor with magnetization and possibly with external magnetic fields. However, an open question arises: Can the Néel tensor also interact with spin currents? Theoretically, when a spin current is injected through a polycrystalline AFM, its interaction with the Néel tensor might lead to modifications in the spin current itself, described by:

(8) $$\Delta J_{i}^{\alpha }=A\sum_{\beta }N^{\alpha \beta }J_{i}^{\beta }\;$$

The change of the spin current arises from the contraction between the Néel tensor *N* ^αβ^ and the spin current $J_{i}^{\beta }$, and the overall strength *A*. This equation suggests that the Néel tensor could serve as a spin current filter, selectively modifying the spin polarization based on local AFM spin correlations. This concept also prompts a reciprocal question: Can the spin current, in turn, influence the Néel tensor? If valid, this would offer an alternative means of dynamically controlling the Néel tensor orientation, expanding the tunability of AFM spintronics.

The demonstrated training and memory effects of the Néel tensor torque introduce a programmable degree of control in spintronic devices. Unlike conventional exchange bias, which is fixed during fabrication, the hidden Néel tensor behind exchange bias offers a reconfigurable platform for field‐free SOT switching. This dynamic tunability has profound implications for several emerging technologies, for example, field‐free SOT‐MRAM, neuromorphic computing, and physically unclonable functions (PUFs). By bridging fundamental physics with practical applications, the findings in this work establish the Néel tensor as a powerful new degree of freedom in AFM spintronics, paving the way for novel device architectures and adaptive, programmable magnetic systems.

## 4 Conclusion

In conclusion, this study establishes the Néel tensor as a groundbreaking framework for understanding spin configurations in polycrystalline AFMs, surpassing the conventional Néel vector. Using a machine‐learning algorithm, we demonstrate how the Néel tensor captures statistical spin correlations and the resultant Néel tensor torque enables field‐free SOT switching in Pt/Co/IrMn trilayers with trainable polarity. Our findings bridge theory, experiments, and applications, paving the way for reconfigurable spintronic memory, neuromorphic computing, and hardware security via physically unclonable functions.

## 5 Experimental Section

A multilayer film stack of Si/SiO_2_/Ta(2)/Pt(5)/Co(1)/IrMn(8)/Ti(3) (thickness in nanometers) was fabricated using magnetron sputtering in a vacuum chamber with a base pressure of 4 × 10^−8 ^Torr. The 2 nm Ta layer served as an adhesion layer for Pt deposition and promoted the (111) texture, which facilitated the perpendicular magnetic anisotropy of the Co layer. To ensure uniform deposition, the substrate was continuously spun during the sputtering process. The magnetic properties of the as‐deposited films were characterized using a vibrating sample magnetometer (VSM) at 300 K.

For transport measurements, the films were patterned into a Hall bar device with dimensions of 10 µm in width and 50 µm in length. A Ta(10)/Pt(100) electrode was deposited via the lift‐off technique. Anomalous Hall effect (AHE) measurements were conducted to obtain the hysteresis loops by applying a direct current (DC) of 1 mA along the longitudinal channel under a perpendicular magnetic field (*H_z_​*), while the transverse voltage was recorded. SOT switching was performed using a pulse current (*I_Write_* ​) of varying amplitudes, with an in‐plane field (*H_x_* ​) collinear to the current channel of the device. After applying a SOT pulse of 0.3 ms duration, a 1 mA DC was used to measure the AHE signal (*R_xy_​*) to track the magnetization state. For the field‐setting experiment, the samples were loaded into a Physical Properties Measurement System (PPMS) with the highest field of 7 Tesla applied toward the y‐direction of the devices. Then, the field‐free SOT switching was performed to verify the switching polarity. The polarity resetting was performed using a rapid thermal annealing facility to thermally treat the samples at 200 °C for 2 min.

## Conflict of Interest

The authors declare no conflict of interest.

## Author Contributions

C.H.L. and H.H.L. planned and supervised the project. C.Y.Y. carried out SOT measurements and data analysis. C.H.T. and S.H.C. finished the thin film deposition and device fabrication. H.H.L. developed the theoretical framework and finished the calculations. H.H.L. and C.H.L. wrote the manuscript, and all authors discussed the results together.

## Supporting information



Supporting Information

## Data Availability

The data that support the findings of this study are available in the supplementary material of this article.
