# Supplementary material for: Néel Tensor Torque in Polycrystalline Antiferromagnets
Source: Adv Mater. 2025 Aug 29;38(9):e06462. doi: 10.1002/adma.202506462 (PMC12902605; doi:10.1002/adma.202506462)
Supplement: Supplementary file 1 — Supporting Information [file ADMA-38-e06462-s001.docx]

**(Supplementary information)**

**Néel tensor torque in polycrystalline antiferromagnets**

Chao-Yao Yang, Sheng-Huai Chen_,_ Chih-Hsiang Tseng, Hsiu-Hau Lin*, Chih-Huang Lai*

Chao-Yao Yang, Sheng-Huai Chen_,_ Chih-Hsiang Tseng, and Chih-Huang Lai

Department of Materials Science and Engineering, National Tsing Hua University, Hsinchu, 300044, Taiwan.

Chao-Yao Yang

Department of Materials Science and Engineering, National Yang Ming Chiao Tung University, Hsinchu, 300093, Taiwan.

Center for Emergent Functional Matter Science, National Yang Ming Chiao Tung University, Hsinchu 300093, Taiwan

Hsiu-Hau Lin

Department of Physics, National Tsing Hua University, Hsinchu 300044, Taiwan.

College of Semiconductor Research, National Tsing Hua University, Hsinchu 300044, Taiwan

Chih-Huang Lai

College of Semiconductor Research, National Tsing Hua University, Hsinchu 300044, Taiwan

Corresponding Authors:

Prof. Hsiu-Hau Lin

E-mail: [hsiuhau.lin@phys.nthu.edu.tw](mailto:hsiuhau.lin@phys.nthu.edu.tw)

Prof. Chih-Huang Lai

E-mail: [chlai@mx.nthu.edu.tw](mailto:chlai@mx.nthu.edu.tw)

*Keywords: Spin-orbit torque, Neel tensor torque, Field-free switching, Physically Unclonable functionality*

**Supplementary Information 1 – Exclusion of the in-plane exchange bias**


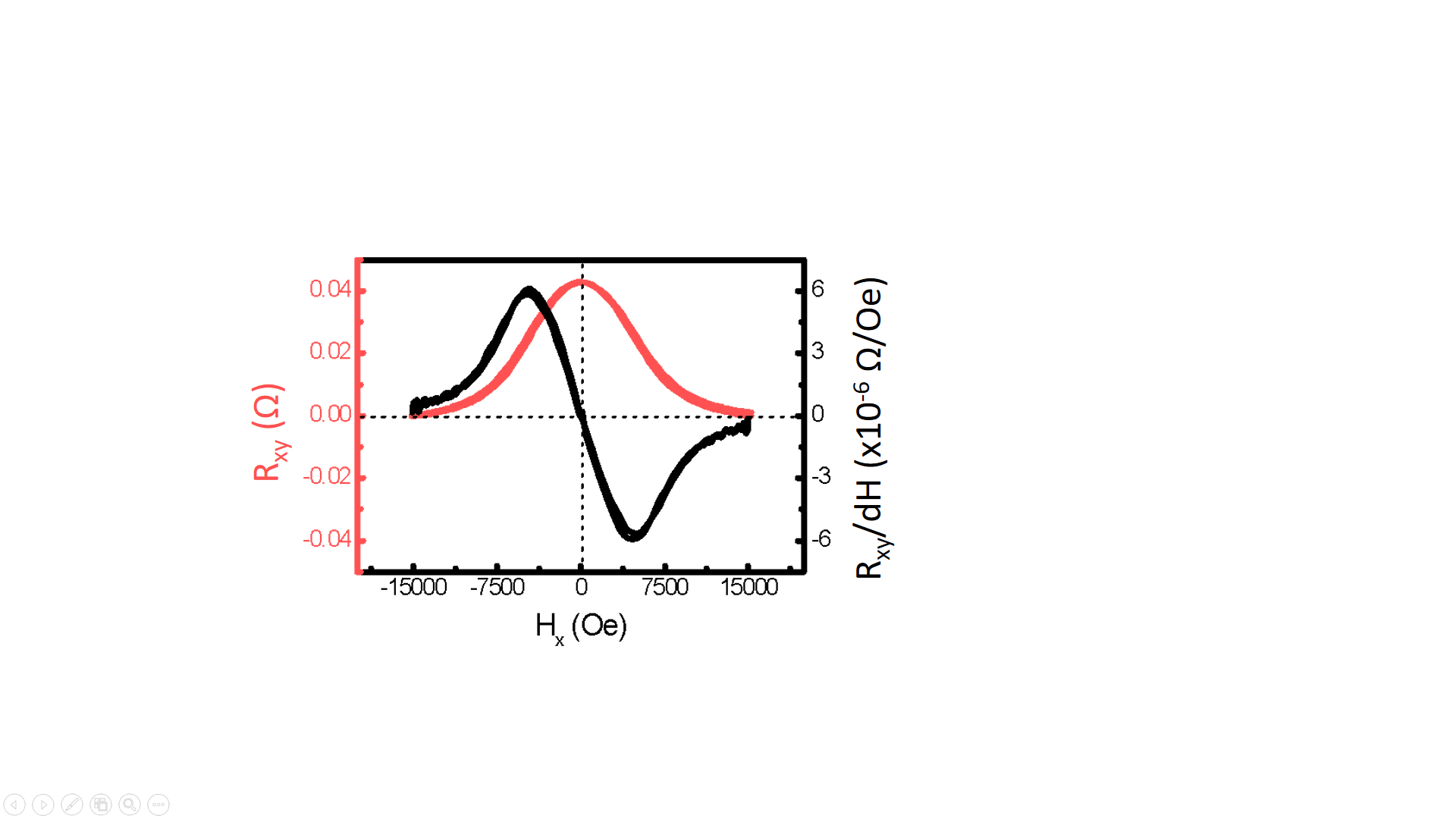


**Figure S1｜Absence of in-plane exchange bias.** Anomalous Hall effect ($R_{xy}$) versus $H_{x}$ curve (red) and the first-order derivative curve (black) taken after performing the SOT switching. The centrosymmetric feature on the $R_{xy}$-$H_{x}$ curve suggests the absence of exchange bias developed along the longitudinal direction (*x*-direction) of the device. The derivative $dR_{xy}/dH_{x}$ curve (black) is also shown across the origin to ensure the claim.

**Supplementary Information 2. – Intrinsic polarity and “switching ratio”**


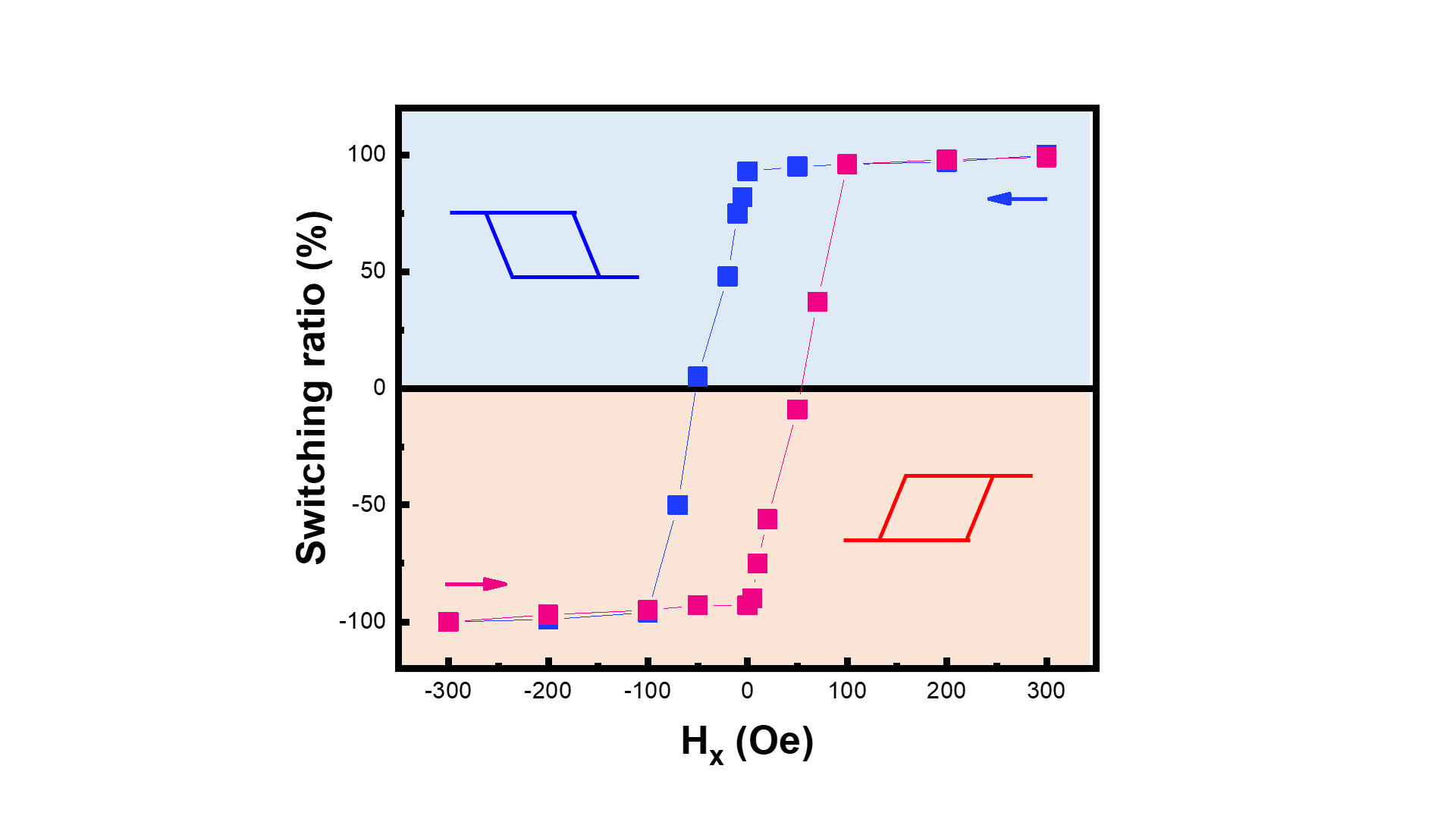


**Figure S2 ｜SOT switching ratio versus** $\boldsymbol{H}_{\boldsymbol{x}}$**.** Blue and red curves are obtained from two as-fabricated Pt/Co/IrMn devices and performed from the $H_{x}$-assisted switching as arrowed. The “switching ratio” is defined by the change of $R_{xy}$ on the SOT switching curve relative to that taken with $H_{x}=300$ Oe. The blue (red) curve is taken by performing SOT with $H_{x}=300$ Oe (-300 Oe) first and then subsequently performing SOT with various $H_{x}.$Note that the polarity of the blue and red curves stands for the SOT switching of $P=-1$ and $P=+1$, respectively. Two important characteristics should be noticed: the magnitudes of the switching ratio at $H_{x}=0$Oe for both cases are still larger than 90 %, suggesting the robust field-free switching shown in **Figure 1(e)** and **Figure 1(f)** in the main text. Furthermore, once the SOT switching was performed from positive $H_{x}$, the switching ratio versus $H_{x}$ correlation is determined as a fingerprint, as shown by the blue plots. The polarity of the field-free switching is always $P=-1$. After being set by $H_{x}=300$ Oe (blue curve), even if we applied a small negative$H_{x}$ ($H_{x}\geq-50 Oe)$, the polarity remains the same, indicating the Néel tensor torque prevails over the field torque under these conditions.

**Supplementary Information 3 – Sign rules from the Landau-Lifshitz-Gilbert equation**

The polarity of the SOT switching is dictated by several important sign rules and can be derived from the Landau-Lifshitz-Gilbert (LLG) equation for the FM magnetization,

$$\frac{\partial\hat{m}}{\partial t}=\alpha\hat{m}\times\frac{\partial\hat{m}}{\partial t}-\gamma\hat{m}\times\vec{H}_{\text{eff}}- \gamma\sigma C_{J} \hat{m}\times\left( \hat{m}\times\hat{y} \right) (S1)$$

where $\hat{m}=\left( m_{x},m_{y},m_{z} \right)$ is the unit vector of the magnetization, $\alpha$ is the damping constant and $\gamma$ represents the gyromagnetic ratio. Here $\sigma=+1, -1$ denotes that the spin current is injected from the top or bottom layer relative to the FM layer, respectively. Two types of torques are present: the usual torque from the effective magnetic field $\vec{H}_{\text{eff}}$ and the Slonczewski torque with strength denoted by the parameter $C_{J}=\frac{\hbar}{2e}\frac{\theta_{\text{SH}}}{M_{s}t_{F}} J$, where $J$ is the current density,$\theta_{\text{SH}}$ is spin Hall angle, $t_{F}$ is the thickness of the FM layer and $M_{s}$ is the saturation magnetization.

The magnetization vector $\hat{m}$ quickly damps into the steady state ($\partial\hat{m}/\partial t=0$) with direction determined by the torque balance between the longitudinal field and the spin-orbit interaction. The dynamics of the magnetization can be classified by a current density threshold,

$$J_{c}=\frac{e}{\hbar}\frac{M_{s}t_{F}}{\theta_{\text{SH}}}\left( H_{K}-\sqrt{2}H_{x} \right)$$

When the current density is below the threshold $J<J_{c}$, the magnetization remains in the initial direction. Above the current density threshold $J>J_{c},$ the torque balance gives rise to the stationary state $\hat{m}^{*}=\left( m_{x}^{*},m_{y}^{*},m_{z}^{*} \right)$,

$$m_{x}^{*}= 0 (S2)$$

$$m_{y}^{*}=\frac{1}{\sigma C_{J}}\sqrt{\sigma^{2}C_{J}^{2}-H_{x}^{2}} (S3)$$

$$m_{z}^{*}=\frac{H_{x}}{\sigma C_{J}} (S4)$$

The stationary magnetization $\vec{M}^{*}=M\hat{m}^{*}=M(0,m_{y}^{*},m_{z}^{*})$. For realistic parameters, $M_{z}^{*}\ll M_{y}^{*}$, i.e., $\vec{M}^{*}$ is very close to the $y$-axis with a small tilting angle $\theta_{t}$ away from the $x-y$ plane,

$$\theta_{t}= \frac{\pi}{2}-\theta=\sin^{-1} \left( \frac{H_{x}}{{\sigma C}_{J}} \right)$$

From the steady-state solution $\vec{M}^{*}=(0,M_{y}^{*},M_{z}^{*})$, we are now ready to derive the sign rules for the stationary magnetization during the pulse-ON period,

$$\text{sign}\left( M_{y}^{*} \right)=\sigma\text{sign}\left( C_{J} \right)=\sigma\text{sign}\left( \theta_{\text{SH}} J \right) (S5)$$

$$\text{sign}\left( M_{z}^{*} \right)=\sigma\text{sign}\left( H_{x}C_{J} \right)=\sigma\text{sign}\left( H_{x}\theta_{\text{SH}} J \right) (S6)$$

**Supplementary Information 4 – Polarity of SOT switching**

The polarity is closely related to the symmetry breaking in the SOT switching. Below the current threshold ($J<J_{c}$), the magnetization $\vec{M}$ in the FM layer remains in the vicinity of the $z$-axis, while it gets pushed toward the $y$-axis when the current exceeds the threshold ($J>J_{c}$). In the presence of a longitudinal magnetic field, the Landau-Lifshitz-Gilbert (LLG) equation gives rise to a temporary stationary solution (during the pulse-on period) for the magnetization $\vec{M}^{*}=(0,M_{y}^{*},M_{z}^{*})$ where $M_{z}^{*}\ll M_{y}^{*}$, satisfying the following sign rules,

$$\text{sign}\left( M_{y}^{*} \right)=\sigma\text{sign}\left( \theta_{\text{SH}} J \right) (S5)$$

$$\text{sign}\left( M_{z}^{*} \right)=\sigma\text{sign}\left( H_{x}\theta_{\text{SH}} J \right) (S6)$$

Here $\sigma=+1, -1$ denotes that the spin current is injected from the top or bottom layer relative to the FM layer, respectively. As elaborated below, these sign rules pave the first step to understanding why symmetry breaking is necessary for SOT switching.

Define the polarity of the SOT switching as $P\equiv\text{sign}\left( M_{z} J \right)$. When the pulse is off, the magnetization relaxes from $M_{z}^{*}$ to the final value $M_{z}$ (both share the same sign) so that $P\equiv\text{sign}\left( M_{z} J \right)=\text{sign}\left( M_{z}^{*} J \right)$. Making use of the relation in (S5), the polarity can be rewritten in a more suggestive form,

$$P=\sigma\text{sign}\left( \theta_{\text{SH}} \right)\cdot\text{sign}\left( M_{y}^{*}M_{z}^{*} \right) (S7)$$

In the above, we insert the trivial identity $\text{sign}\left( M_{y}^{*}M_{y}^{*} \right)=1$ to facilitate the derivation. The first factor is related to the spin current injection, depending on the setup geometry $\sigma$ and the materials property $\theta_{\text{SH}}$. The second factor is the sign of the product $M_{y}^{*}M_{z}^{*}$, describing how parity symmetry is broken during the pulse-ON period.

Combining the sign rules $M_{y}^{*}$ and $M_{z}^{*}$ in Eq.(S5) and Eq. (S6) derived from the LLG equation leads to the important relation concerning symmetry breaking,

$$\text{sign}\left( M_{y}^{*}M_{z}^{*} \right)=\text{sign}\left( H_{x} \right) (S8)$$

It is rather remarkable that the sign of the product $M_{y}^{*}M_{z}^{*}$ solely depends on the external magnetic field $H_{x}$. Therefore, in the presence of the external magnetic field $H_{x}$, the polarity of the SOT switching is

$$P=\sigma\text{sign}\left( H_{x}\theta_{\text{SH}} \right) (S9)$$

Note that the above formula reveals the necessity of symmetry breaking for the SOT switching – the polarity $P$ is ill-defined for $H_{x}=0$.

Applying the sign rule to the experimental setup described in **Figure 1**, the spin current is injected from the bottom layer so that $\sigma=-1$. The sign of spin Hall angle $\theta_{\text{SH}}$ (Pt layer) is positive. When the longitudinal field $H_{x}$ is pointing to the positive $x$-direction, the polarity $P=\sigma\text{sign}\left( H_{x}\theta_{\text{SH}} \right)=-1$, consistent with the experimental finding from the $M-I$ curve. In fact, the above sign rule is verified in all our experimental results for the SOT switching.

It is important to emphasize that, after the dynamical setting, the device exhibits field-free SOT switching with an intrinsic polarity. Once the polarity is set, it remains robust and will not be erased by applying the opposite field, as shown in **Figure 1 (e) and (f)**. The field-free SOT switching found in our experiment is a strong hint for some unknown symmetry-breaking interaction, later identified as the Néel tensor torque.

**Supplementary Information 5 – AFM viewed as a probability distribution of spin orientations**

As explained in the main text, an AFM domain can be viewed as the statistical ensemble of three correlated random variables $s^{x}$, $s^{y}$, $s^{z}$ with specific probability distribution depending on the microscopic spin-dependent interactions. Viewing the AFM domain as the ensemble, the statistical average can be expressed in terms of summation over all spins,

$$\left\langle O \right\rangle=\frac{1}{n_{d}}\sum_{i=1}^{n_{d}} O_{i} (S10)$$

Here, 𝑂 denotes an observable depending on the random variables 𝑠^𝛼^, and 𝑂_𝑖_ is a specific realization from the statistical ensemble. For simplicity, let us assume the residual spin $\vec{S}_{R}=0$, so that the correlation matrix $\boldsymbol{Q}$, capturing the correlations between three spatial components of the spin, is simplified,

$$Q^{\alpha\beta}\equiv\left\langle s^{\alpha}s^{\beta} \right\rangle=\frac{1}{n_{d}}\sum_{i=1}^{n_{d}} s_{i}^{\alpha}s_{i}^{\beta}=\frac{1}{n_{d}}N^{\alpha\beta} (S11)$$

It is rather interesting to observe that the correlation matrix $\boldsymbol{Q}$ from a statistical perspective is just the Néel tensor $N$ defined before (up to a normalization factor $n_{d}$). By rotation from the lab frame to the principal frame, the correlation matrix (and also the Néel tensor) is diagonalized,

$$\boldsymbol{Q}^{\boldsymbol{'}}=\left( \begin{matrix} \Delta_{x'} & 0 & 0 \\ 0 & \Delta_{y'} & 0 \\ 0 & 0 & \Delta_{z'} \end{matrix} \right) (S12)$$

In the principal frame, the spin components $s^{x'}$, $s^{y'}$, $s^{z'}$ are no longer correlated because the off-diagonal elements of $\boldsymbol{Q}^{\boldsymbol{'}}$ are identically zero. The spin arrangement viewed in the principal frame is thus characterized by the variances $\Delta_{x'}$, $\Delta_{y'}$, $\Delta_{z'}$ along three principal axes. Note that “uncorrelated” is not the same as “independent” in data science and our analysis here resembles the Principal Components Analysis (PCA) within the linear factor model in machine learning and shall not be mistaken as Independent Components Analysis (ICA).

The statistical perspective provides an alternative way to look at spin arrangements in an AFM domain. Multiplying the normalization factor $n_{d}$, the eigenvalues of the Néel tensor are $n_{d}\Delta_{x'}$, $n_{d}\Delta_{y'}$, $n_{d}\Delta_{z'}$ respectively. In the principal frame, the spin components are uncorrelated and the spin variance $\Delta_{\alpha'}$ renders an intuitive picture for the spin arrangement. As shown in **Figure 3(a)**, in a collinear AFM along the $z'$-axis, spin variance $\Delta_{z'}=s^{2}$ (long axis) while those in the transverse directions are zero, $\Delta_{x'}=\Delta_{y'}=0$ (short axes). In a non-collinear AFM shown in **Figure 3(c)**, the spin variances $\Delta_{z'}>\Delta_{y'}>\Delta_{x'}$, so that the spin arrangement contains largest weight along the $z'$-axis (long axis) and the smallest weight along the $x'$-axis (short axis).

**Supplementary Information 6 – Néel tensor torque**

The Néel tensor torque provides another way to understand the field-free SOT switching with intrinsic polarity. In the following, we would like to show that the intrinsic polarity satisfies the sign rule,

$$P=\sigma\text{sign}\left( \theta_{\text{SH}} \right)\cdot\text{sign}\left( n_{y}^{*}n_{z}^{*} \right) (S13)$$

where $\vec{n}^{*}=(0,n_{y}^{*},n_{z}^{*})$ corresponds to the short axis of the Néel tensor aligned in the $y$-$z$ plane. For example, if the short axis of the Néel tensor is aligned in the 1^st^ and 3^rd^ quadrants of the $y$-$z$ plane, as shown in **Figure 4(d)**, the intrinsic polarity is

$$P=\sigma\text{sign}\left( \theta_{\text{SH}} \right)\cdot\text{sign}\left( n_{y}^{*}n_{z}^{*} \right)=\left( -1 \right)\cdot1= -1$$

The combined effects from the Slonczewski and Néel tensor torques give rise to the above sign rule. When the Slonczewski torque drives the magnetization to the positive $y$ direction, the Néel tensor torque pushes it upward, and the stationary magnetization $\vec{M}^{*}=(0,M_{y}^{*},M_{z}^{*})$ lies in the 1^st^ quadrant of the $y$-$z$ plane, as shown in **Figure 4(d)**. When the Slonczewski torque drives the magnetization to the negative $y$ direction, the Néel tensor torque pushes it downward with stationary magnetization $\vec{M}^{*}$ in the 3^rd^ quadrant. In consequence, the Néel tensor torque leads to the sign rule,

$$\text{sign}\left( M_{y}^{*}M_{z}^{*} \right)=\text{sign}\left( n_{y}^{*}n_{z}^{*} \right)=1$$

Making use of the sign rule derived in the previous paragraphs, the intrinsic polarity of the SOT switching is

$$P=\sigma\text{sign}\left( \theta_{\text{SH}} \right)\cdot\text{sign}\left( M_{y}^{*}M_{z}^{*} \right)=\sigma\text{sign}\left( \theta_{\text{SH}} \right)\cdot\text{sign}\left( n_{y}^{*}n_{z}^{*} \right)= -1$$

The sign rule reflects the short axis of the Néel tensor $\vec{n}^{*}=(0,n_{y}^{*},n_{z}^{*})$ is set by the stationary magnetization $\vec{M}^{*}=(0,M_{y}^{*},M_{z}^{*})$ during the field-SOT setting or the field setting. The sign rule is readily generalized to the external field and the exchange bias as well. As shown in **Figure 6(a)**, the external field $\vec{H}=(0,H_{y},0)$ and the exchange bias  $\vec{H}_{b}=(0,0,H_{b})$ replace the role of $n_{y}^{*}$ and $n_{z}^{*}$ in Eq. (S13) respectively so that the sign rule now takes the form,

$$P=\sigma\text{sign}\left( \theta_{\text{SH}} \right)\cdot\text{sign}\left( H_{y}H_{b} \right)$$

It agrees with the experimental findings as elaborated in the maintext.

**Supplementary Information 7 – Néel tensor for tetrahedral spin arrangement**

In our polycrystalline IrMn film, its $\left\langle111 \right\rangle$ direction is aligned along the $z$-axis while the transverse directions are randomly oriented. The microscopic spin-dependent interaction leads to the so-called 3Q tetrahedral spin arrangement, as shown in Figure **S3**. In an ideal situation, all spins at the corners point to the body center of the tetrahedron,

$$\vec{s}_{1}=s\left( 0,0,-1 \right)$$

$$\vec{s}_{2}=s\left( -\frac{2\sqrt{2}}{3},0,\frac{1}{3} \right)$$

$$\vec{s}_{3}=s\left( \frac{\sqrt{2}}{3},-\frac{\sqrt{6}}{3},\frac{1}{3} \right)$$

$$\vec{s}_{4}=s\left( \frac{\sqrt{2}}{3},\frac{\sqrt{6}}{3},\frac{1}{3} \right)$$

The Néel tensor can be computed by summing over all spin contributions. For the ideal tetrahedral spin arrangement, the Néel tensor turns out to be zero,

$$N^{\alpha\beta}=\sum_{i=1}^{4} s_{i}^{\alpha}s_{i}^{\beta}=0 (S14)$$

Thus, the 3Q tetrahedral spin arrangement is sensitive to all perturbations, leading to non-zero Néel tensors. However, for realistic materials below the blocking temperature, the Néel tensor associated with each domain is likely to be non-vanishing due to domain-domain interaction or/and other microscopic spin-dependent interactions, distorting the spin arrangement from the ideal 3Q tetrahedral one.


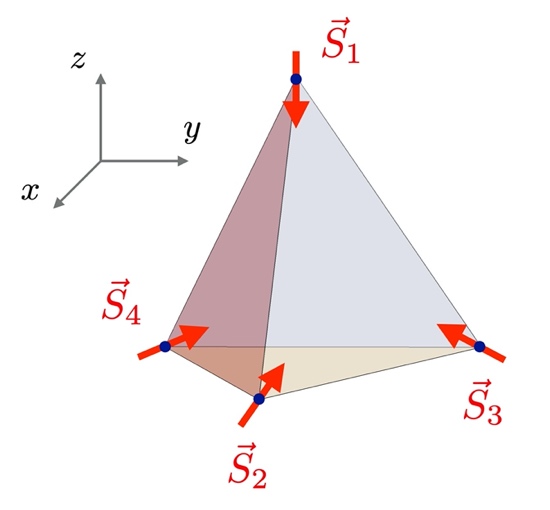


**Figure S3｜Tetrahedral spin arrangement.** In our polycrystalline IrMn film, its $\left\langle111 \right\rangle$ direction is aligned perpendicularly and the AFM coupling leads to the so-called 3Q tetrahedral spin arrangement. It is straightforward to compute the Néel tensor for the ideal tetrahedral spin arrangement and the answer turns out to be zero.
